# The Erogenous Mirror: Intersubjective and Multisensory Maps of Sexual Arousal in Men and Women

**DOI:** 10.1007/s10508-020-01756-1

**Published:** 2020-06-12

**Authors:** Lara Maister, Aikaterini Fotopoulou, Oliver Turnbull, Manos Tsakiris

**Affiliations:** 1 School of Psychology, College of Human Sciences, Prifysgol Bangor University, Gwynedd, Wales, LL57 2AS UK; 2 Department of Psychology, Royal Holloway University of London, Egham, Surrey, UK; 3 Department of Clinical, Educational and Health Psychology, University College London, London, UK; 4 The Warburg Institute, School of Advanced Study, University of London, London, UK; 5 Department of Behavioural and Cognitive Sciences, Faculty of Humanities, Education and Social Sciences, University of Luxembourg, Esch-sur-Alzette, Luxembourg

**Keywords:** Erogenous zone, Touch, Gender, Multisensory, Vision, Sexual arousal

## Abstract

Erogenous zones of the body are sexually arousing when touched. Previous investigations of erogenous zones were restricted to the effects of touch on one’s own body. However, sexual interactions do not just involve being touched, but also involve touching a partner and mutually looking at each other’s bodies. We take a novel interpersonal approach to characterize the self-reported intensity and distribution of erogenous zones in two modalities: touch and vision. A large internet sample of 613 participants (407 women) completed a questionnaire, where they rated intensity of sexual arousal related to different body parts, both on one’s own body and on an imagined partner’s body in response to being touched but also being looked at. We report the presence of a multimodal erogenous mirror between sexual partners, as we observed clear correspondences in topographic distributions of self-reported arousal between individuals’ own bodies and their preferences for a partner’s body, as well as between those elicited by imagined touch and vision. The erogenous body is therefore organized and represented in an interpersonal and multisensory way.

**Electronic supplementary material:**

The online version of this article (10.1007/s10508-020-01756-1) contains supplementary material, which is available to authorized users.

## Introduction

From an evolutionary perspective, sexual arousal is thought to be primarily elicited by tactile stimulation of the genitals (Gallup, Towne, & Stolz, 2018). However, during sexual interaction, human partners often mutually caress other body parts that have no anatomical links to the genitals. Several of these extra-genital erogenous zones are capable of eliciting sexual arousal when stimulated, sometimes even eliciting orgasm (Younis, Fattah, & Maamoun, 2016). These areas can encompass up to 26% of the body surface (Nummenmaa, Suvilehto, Glerean, Santtila, & Hietanen, 2016) and are reported as arousing in the large majority of individuals (Younis et al., 2016). To date, only a small number of studies (Nummenmaa et al., 2016; Turnbull, Lovett, Chaldecott, & Lucas, 2014) have systematically mapped the tactile erogenous zones of the body. Across these studies, several extra-genital body parts were reliably identified as capable of eliciting high levels of sexual arousal: these included the breasts, nipples, lips, neck and nape of neck, ears, buttocks, and inner thigh (Nummenmaa et al., 2016; Turnbull et al., 2014; Younis et al., 2016). The topographical distribution and intensity of erogenous zones are similar for men and women (Nummenmaa et al., 2016; Turnbull et al., 2014) and are relatively impervious to sociological, demographic, and cultural factors (Turnbull et al., 2014).

An important feature of erogenous zones is their interpersonal function. Nummenmaa et al. (2016) mapped erogenous zones in two different situations, by asking participants to rate the sexual arousal elicited by tactile stimulation of different body parts when having sex with a partner and while masturbating. A significantly larger overall area of the body surface was rated as erogenous during sex than during masturbation. This suggests that the stimulation of extra-genital erogenous zones may play a more important role in interpersonal contexts, perhaps serving a pair-bonding function, via stimulation of C-tactile fibers known to convey both pleasant, sensual touch (Dunbar, 2010; Morrison, Bjornsdotter, & Olausson, 2011) and erotic sensations (Bendas et al., 2017; Jönsson et al., 2015), or via other top-down factors (Gentsch, Panagiotopoulou, & Fotopoulou, 2015).

Notwithstanding the advances that the aforementioned studies have brought to the study of tactile organization of erogenous zones, we highlight three key limitations that we aimed to address in our study. First, given the interpersonal nature of extra-genital stimulation during sexual activity, it seems surprising that erogenous zones have only been considered from the reference point of the individual’s own body. Individuals not only become aroused when they are touched in certain areas, but are also likely to find touching certain areas of their partner’s body arousing, although this has never been systematically investigated. Therefore, erogenous zones can be mapped both with reference to ourselves, i.e., which parts of our own body we find arousing when touched, but also with reference to a partner, i.e., which areas of our partner’s body we find arousing to touch. In both cases, the maps reflect our own experiences of arousal, but differ with regard to the body that the map is spatially referenced to. Importantly, a correspondence between the topography of erogenous zones on our own body and the topography of what we find erogenous to touch on a partner’s body would suggest that we may have an interpersonal somatotopic map of erogenous zones, providing a direct self-other “mirroring” of erogenous experience. We therefore aimed to systematically characterize, for the first time, the topography of one’s own erogenous zones of a partner’s body, as well as test for the existence of an “erogenous mirror.”

Second, what we know of the distribution and function of erogenous zones stems from the restriction of recent investigations to the tactile modality. As most experiences, sexual arousal is not a unisensory experience. Although touch plays a primary role in the majority of sexual interactions, they are also regularly characterized by visual stimulation, in the form of looking at our partner’s body and our partner looking at our body. It is also known that visual stimuli of bodies can elicit intense sexual arousal in the absence of tactile stimulation (Kühn & Gallinat, 2011; Redouté et al., 2000). Importantly, there are intriguing gender differences in arousal induced by visual sexual stimuli (Bolmont, Pegna, & Bianchi-Demicheli, 2017; Lykins, Meana, & Strauss, 2008; Rupp & Wallen, 2008), evident in differential gaze fixation patterns to erotic images (Rupp & Wallen, 2008). We therefore extended our investigation of erogenous zones to map patterns of arousal induced by visual stimulation as well as tactile stimulation, for both women and men, and expected to find some gender differences in the effects of modality on the distribution of these maps. Specifically, we aimed to investigate the potential similarities and differences between modalities with regard to erogenous maps. A correspondence between the tactile and visual erogenous maps would suggest that our somatotopic map of erogenous zones may be bimodal rather than unimodal, activated by both visual and tactile information, similar to mirror systems in other domains. Therefore, our second aim was to test for the presence of a “multimodal correspondence map.”

Third, although previous studies have highlighted the close correlation between the topography of male and female erogenous zones, it is as yet unknown how men’s and women’s sexual preferences align with each other. By mapping erogenous zones with reference to both one’s own and a partner’s body, we have the unique opportunity to develop a “Mutual Pleasure Index,” to investigate whether one gender’s preferences for an opposite-gender partner’s body aligns with that opposite-gender’s preferences for their own body. In other words, we can investigate whether there is any correspondence between the areas that men, on average, find arousing to touch/look at on a woman, and the areas that women on average find arousing to be touched or looked at; and vice versa, whether women’s preferences for where they like to touch or look at men map on to men’s preferences for where they like to be touched or looked at.

To achieve these aims, we used a large, internet-based sample (final *N* = 613) to carry out a systematic mapping of self-reported erogenous zones, assessing the effects of two key factors. The first factor reflected the person’s body the map referred to, either one’s own or a partner’s, and the second factor reflected the modality of stimulation: either touch or vision. In this way, participants rated the erogeneity of 41 body parts, on a scale from 0 (not at all arousing) to 10 (extremely arousing) in four distinct contexts: (1) when they were touched (own-body/touch condition); (2) when they touched their partner (partner-body/touch condition); (3) when they were looked at (own-body/look condition); and (4) when they looked at their partner (partner-body/look condition).This design enabled us to investigate the effects of the target body and modality factors, both singly and in interaction, on arousal, as well as investigate correspondences between the erogenous maps produced in each of the four contexts (allowing us to achieve our three central aims). Finally, by including several important individual difference variables, we were able to examine the effects of gender, relationship status, sexual satisfaction, and perceived sensuality on our ratings.

## Method

### Participants

Responses were collected online. The questionnaire was advertised across three university campus notice boards and on social media. As incentive, participants were entered into a prize draw to win one of two Apple iPad Minis if they reached the end of the questionnaire and entered their email address. There were no specific eligibility criteria. The study was approved by the Royal Holloway University of London Psychology Ethics Committee.

In total, 613 completed questionnaires were obtained (407 women, *M*_Age_ = 28.8 years (SD = 9.8), 206 men, *M*_Age_ = 33.9 years (SD = 12.9), further demographic information is shown in Table 1). The attrition rate (unfinished questionnaires, *N* = 204) was 25%. This reflected incomplete questionnaires (*N* = 195), as well as questionnaires where all responses were the same (where an individual had put a response of 10 for all body parts for all questions, for example). While these responses may have been genuine in a small number of cases, we felt that they were more likely to be reflecting improper participation and were removed (*N* = 9).

**Table 1:** Demographics of study samples of women and men

	Women (*N* = 407)		Men (*N* = 206)	
Factor	*N*	%	*N*	%
*Sexual orientation*				
Heterosexual	335	82.3	166	80.6
Bisexual/pansexual	57	14.0	16	7.8
Homosexual	11	2.7	24	11.7
Asexual	1	0.2	0	0.0
Declined	3	0.7	0	0.0
*Relationship status*				
Single	115	28.3	60	29.1
In a relationship	280	68.8	140	68.0
Declined	12	2.9	6	2.9
*Ethnic origin*				
White	340	83.5	171	83.0
Mixed/multiple ethnic backgrounds	19	4.7	12	5.8
Asian/Asian British	19	4.7	13	6.3
Black/African/Caribbean/Black British	11	2.7	6	2.9
Other	13	3.2	2	1.0
Declined	5	1.2	2	1.0

### Materials and Measures

#### Demographic Questions

Participants were asked several questions regarding demographic information and individual differences. For the demographic information, participants were asked about their age, gender, sexual orientation, whether they were currently in a relationship, and whether they were sexually active currently or had been in the past. They were also asked to rate how sexually satisfied they considered themselves to be on a 5-point scale, and how sensual they perceived themselves to be on a 5-point scale. For women, they were also asked if they were using hormonal contraception, and when their last menstrual cycle began (if applicable).

#### Erogenous Zones Questionnaire

Participants were required to rate, on a computer screen, how arousing they found 41 different body areas, 32 at the front of the body and 9 on the back of the body (see Supplemental Information), under four different conditions, which were counterbalanced. The first asked how arousing each part was to be touched on one’s own body (own-body/touch condition). The second asked how arousing it was to touch each part on a partner (partner-body/touch condition). The third asked how arousing each part was to be looked at (own-body/look condition). Finally, the fourth asked how arousing it was to look at each part on a partner (partner-body/look condition). Thus, the main part of the questionnaire had a 2 (Target Body: Own-Body vs. Partner-Body) × 2 (Modality: Touch vs. Look) within-subjects design.

After reading a brief introductory paragraph (see Supplemental Information), participants were informed that they would see an “Arousal Scale” (Turnbull et al., 2014) which consisted of a list of 41 body areas. We chose a non-pictorial method of displaying the body parts, as showing an image of a mannequin may have confounded the visual imagery processes that may have been necessary to perform the task. This was particularly important given we wanted to compare responses based on touch versus vision, as the displaying of a mannequin (cf. Nummenmaa et al., 2016) may have biased responses toward the visual modality.

Participants were asked to rate the level of arousal of each body area on an 11-point scale, where 1 was not at all arousing and 11 was extremely arousing. Exact instructions to participants are presented in the Supplemental Information. Participants repeated this exercise four times, one for each condition (representing the own-body/touch, partner-body/touch, own-body/look, and partner-body/look conditions). The order of conditions was counterbalanced between participants. They were asked to give their responses with reference to their current or past sexual partner(s), and that if they had noted differences between partners, they were to rate their most intense experiences.

#### Dyadic Adjustment Scale (DAS-7)

The DAS-7 (Hunsley, Best, Lefebvre, & Vito, 2001; Sharpley & Cross, 1982) was completed by participants who were currently in a romantic relationship. This scale consisted of seven items measuring relationship quality and happiness. The first three of the items were rated on a 6-point Likert scale (with endpoints of “always agree” to “always disagree”) and asked about the extent of agreement between the respondent and their partner with regard to three items: philosophy of life; aims, goals, and things believed important; and amount of time spent together. The second three items asked how often specific events occur between the respondent and their partner, including a stimulating exchange of ideas; a calm discussion of something together; and working together on a project. These items were rated on a 6-point Likert scale ranging from “all the time” to “never”). The seventh item asked the respondent to rate the degree of happiness, all things considered, of their relationship, on a seven-point scale (with endpoints of “extremely unhappy” to “perfectly happy”).

### Procedure

After reading the study information and providing informed consent, all participants first provided answers regarding demographic variables. This allowed us to ensure the subsequent questionnaire items were specifically tailored to each individual’s demographic. Those participants who were in a romantic relationship were then given the DAS-7. All participants then completed the Erogenous Zones Questionnaire, before being thanked, debriefed, and provided with the opportunity to enter the prize draw.

### Statistical Analysis

Before analysis, all incomplete questionnaires were removed, along with those with no variance in the responses on the Arousal Scale items. The DAS-7 was coded and analyzed according to Hunsley et al. (2001), allowing us to calculate a composite “relationship quality” score. Cronbach’s alpha was calculated as 0.74, which is very similar to that reported by Hunsley et al.

Unless stated otherwise, all analyses were conducted on the full sample of participants, regardless of sexual orientation. Our analyses first focussed on the effects of Modality (Look vs. Touch) and Target Body (Partner-Body vs. Own-Body) on body-part ratings, as well as gender differences in the effects of these two factors, in order to answer our first key question, “How do arousal ratings differ depending on the modality involved (touching or looking), and the body to which the touch or look is directed (one’s own or a partner’s)?”

First, descriptive analysis was carried out on body-part ratings for the four conditions, focussing on gender similarities and differences. Then, a principal components analysis (PCA) was performed on the 41 body parts to reduce them to their underlying components to facilitate further analysis. These resulting body-part components were entered into a repeated-measures ANOVA to assess the effects of target body, modality, and gender on arousal scores.

Second, advanced analyses were then carried out to assess the relationships between the different erogenous maps obtained from the questionnaire. Specifically, these analyses focussed on (1) the correlation between individuals’ erogenous maps for their own versus a partner’s body, answering our second key question regarding interpersonal maps of erogenous zones, (2) the correlation between individual’s erogenous maps for touching versus looking, answering our third key question regarding *bimodal* erogenous maps, and (3) the correlation between one gender’s erogenous maps for an opposite-gender partner’s body, and the opposite gender’s maps for their own body, at a group level, which answered our fourth key question regarding the “Mutual Pleasure Index.” This analysis was done on heterosexual participants only and then repeated for those who answered about a same-gender partner (including both homosexual participants and some bi-/pansexual participants).

Given the relatively large sample size of the study and thus high power, basing our interpretations on significant (*p* < .05) *p* values would have identified many very small effects. To direct our focus onto only the most meaningful results, we therefore chose to only interpret effects that were of medium size or larger. When ANOVA was implemented, any effect with a partial eta-squared value of greater than 0.06 was interpreted. For *t* tests, effects were deemed meaningful when their Cohen’s (1988) *d* value exceeded 0.5. A post hoc power calculation, carried out in G*Power 3.1.9.2, for the repeated-measures 2 × 2 × 2 ANOVA analyses that formed the basis of the Results (assuming the most conservative correlation among repeated measures present in the data, *r* = .22), yielded a power of > 0.99 when seeking effect sizes of greater than *η*_p_^2^ = .06 (equivalent to an *F*(1, 611) of 39.0).

#### Principal Components Analysis

The PCA was conducted on the 41 rated body parts, by pooling arousal responses to all four conditions. A PCA was chosen as the most suitable approach, as opposed to exploratory factor analysis (EFA), as our aim was not to model the measurement of a latent variable per se but instead to reduce our correlated observed variables to a smaller set of important independent composite variables suitable for further analysis. By running the PCA on the pooled data set, we were able to extract the component structure from the data regardless of condition. Orthogonal (varimax) rotation was used to produce independent components, as the correlation between components was not of primary interest. The Kaiser–Meyer–Olkin test was suitably high, 0.975, and Bartlett’s test for sphericity was significant, *χ*^2^ = 77,010.9, *p* < .0001. Visual inspection of scree plot on the resultant eigenvalues indicated that three components would serve as a parsimonious description of the data. Two further components achieved eigenvalues greater than 1.0 (1.18 and 1.09, respectively); however, they appeared after a clear inflection on the scree plot and so were not included in the final model for reasons of parsimony. The resulting three-component solution accounted for 59.8% of the total variance in the data set (see Table S3 for details).

Using Procrustean rotation, Tucker’s phi was then computed for each of the three components for each condition (Lorenzo-Seva & ten Berge, 2006). This allowed us to assess whether the PCA solution extracted from the pooled data was an adequate approximation of each individual condition. Tucker’s phi indicates “superb” agreement between the pooled solution and all four individual condition-specific solutions (for own-body/touch, *Φ* > .97; for own-body/look, *Φ* > .98; for partner-body/touch, *Φ* = .1.0; for partner-body/look, *Φ* > .99), so we were able to conclude that the structure identified by the pooled solution was an appropriate fit for all conditions.

The loading of each body part onto the three components heuristically identified as “sexual,” “sensual,” and “non-arousing” is displayed in Table S4 (Supplemental Information). The extracted coefficients were standardized for each of the three body components, but not across each condition. This allowed the coefficients to be compared across conditions to assess the effects of target body, modality, and gender on arousal ratings. As the average ratings to areas loading on the non-arousing component were very low across conditions (*M *= 3.14, SD = 1.91), these appeared to be the least erogenous areas of the body, and so no further analysis was carried out on this component to avoid difficulties in interpretation. The sensual (*M *= 5.24, SD = 2.33) and sexual (*M *= 6.87, SD = 2.33) components were retained for further analysis.

## Results

**Fig. 1:**
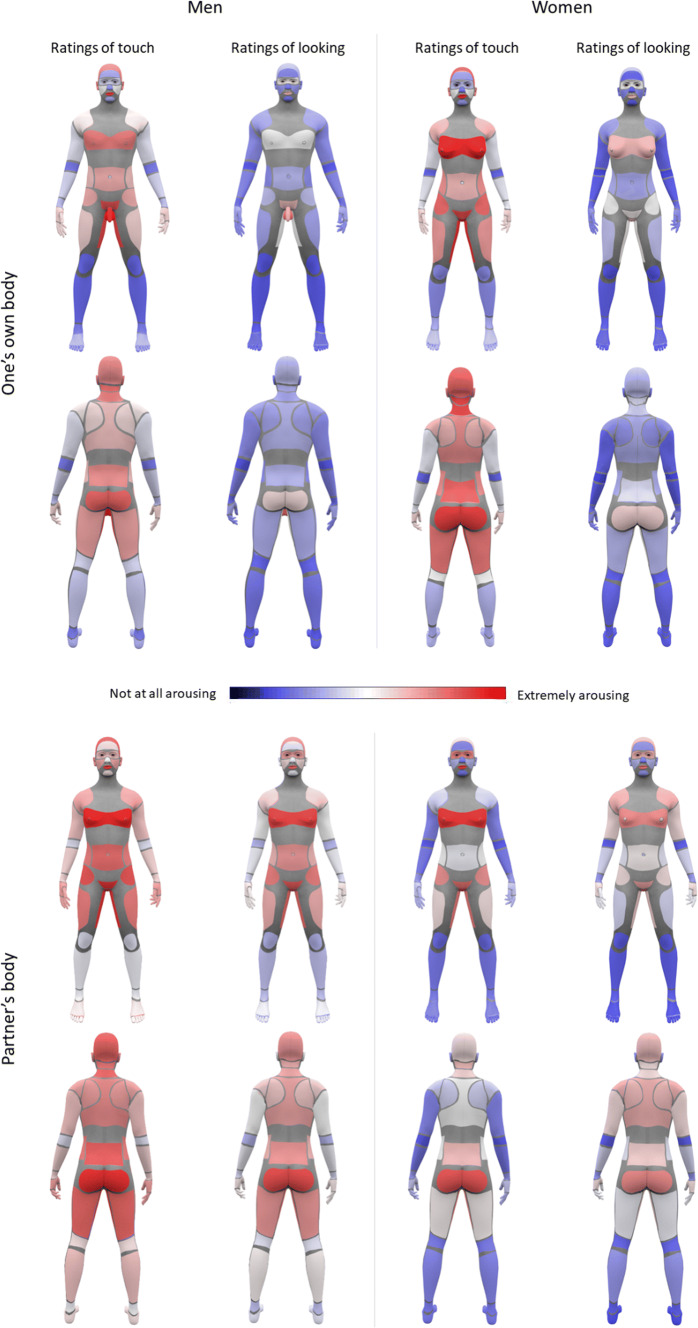
Heat maps representing arousal ratings given to all 41 body parts, split by gender and condition. Participants rated their arousal on a scale of 1 (not at all) to 11 (highly arousing), in four different contexts; (1) when being touched and (2) when being looked at (top panel, illustrated on male or female mannequins matching the gender of the rater), and (3) when touching a partner’s body, and (4) when looking at a partner’s body (bottom panel, all illustrated on androgynous mannequins, as ratings of both men’s and women’s bodies were included). Blue colors denote low levels of arousal, and red colors denote high levels. Women *N* = 407, men *N* = 206 (Color figure online)

Mean ratings for each body part, split by gender, modality, and target body, are visually displayed in Fig. 1, with descriptive data found in the Supplemental Information.

To analyze the arousal ratings, we first ran a PCA on the 41 rated body parts, statistical details of which are provided in the “Method” section. Three components were extracted. The pattern of loadings across all 41 body parts (see Table S3, Supplemental Information) enabled us to make some heuristic interpretations of the three components. The body parts that loaded most heavily onto the first component were all areas that could be defined as stereotypically arousing and generally directly involved in sexual behavior; these included the genitals, breasts, nipples, etc. Therefore, this component was labeled “sexual.” The second component contained body areas that might be considered sensual but not directly sexual, and commonly involved in sensual touch, foreplay, and massage; these included the head, nape of the neck, shoulders, lips, hands, and fingers. This component was therefore labeled “sensual.” Finally, a third component contained body parts that were generally not considered as typically arousing–these included the elbows, knees, chin, calves, etc., and therefore, this component was labeled “non-arousing.” Only the sensual and sexual components were retained for further analysis.

### ANOVA on PCA Components, Assessing Effects of Gender, Modality and Target Body

The two remaining extracted components, for sexual and sensual areas, respectively, were analyzed in separate mixed 2 × 2 × 2 ANOVAs with Target Body (Partner-Body vs. Own-Body) and Modality (Touch vs. Look) as within-subject factors, and gender (man vs. woman) as a between-subjects factor. All statistics are reported with Greenhouse–Geisser correction. For both sexual and sensual areas, two key interactions were revealed; the first, a Gender × Target Body interaction, and the second, a Modality × Target Body interaction. These will be described in turn. All other interactions did not reach effect size criteria to be interpreted, and so will not be discussed further. The full ANOVA results for the sexual areas can be found in Table 2 and for the sensual areas in Table 3.

**Table 2:** ANOVA results for sexual areas

Predictor	*df*_Num_	*df*_Den_	SS_Num_	SS_Den_	*F*	*p*	*η*_p_^2^
(Intercept)	1	611	3.14	1233.22	1.56	.213	.00
Gender	1	611	29.24	1233.22	14.49	< .001	.02*
Modality	1	611	188.46	239.80	480.18	< .001	.44*
Target_Body	1	611	8.27	278.70	18.12	< .001	.03*
Gender × Modality	1	611	0.10	239.80	0.25	.618	.00
Gender × Target_Body	**1**	**611**	**283.41**	**278.70**	**621.31**	**<** **.001**	.50*
Modality × Target_Body	**1**	**611**	**26.76**	**137.53**	**118.91**	**<** **.001**	.16*
Gender × Modality × Target_Body	1	611	2.02	137.53	8.98	.003	.01*

*df*_Num_, degrees of freedom numerator; *df*_Den_, degrees of freedom denominator; SS_Num_, sum of squares numerator; SS_Den_, sum of squares denominator; *η*_p_^2^, partial eta-squared

Asterisks indicate significant effects at *p* < .05; bold text indicates which higher-order effects had effect sizes that were medium-sized or larger

**Table 3:** ANOVA results for sensual areas

Predictor	*df*_Num_	*df*_Den_	*SS*_Num_	*SS*_Den_	*F*	*p*	*η*_p_^2^
(Intercept)	1	611	9.93	1545.04	3.93	.048	.01
Gender	1	611	92.39	1545.04	36.53	< .001	.06*
Modality	1	611	27.13	142.88	116.01	< .001	.16*
Target_Body	1	611	84.80	294.94	175.66	< .001	.22*
Gender × Modality	1	611	0.00	142.88	0.00	.970	.00
Gender × Target_Body	**1**	**611**	**53.81**	**294.94**	**111.46**	**<** **.001**	.15*
Modality × Target_Body	**1**	**611**	**25.92**	**107.34**	**147.53**	**<** **.001**	.19*
Gender × Modality × Target_Body	1	611	0.76	107.34	4.32	.038	.01*

*df*_Num_, degrees of freedom numerator; *df*_Den_, degrees of freedom denominator; SS_Num_, sum of squares numerator; SS_Den_, sum of squares denominator; *η*_p_^2^, partial eta-squared

Asterisks indicate significant effects at *p* < .05; bold text indicates which higher-order effects had effect sizes that were medium-sized or larger

**Fig. 2:**
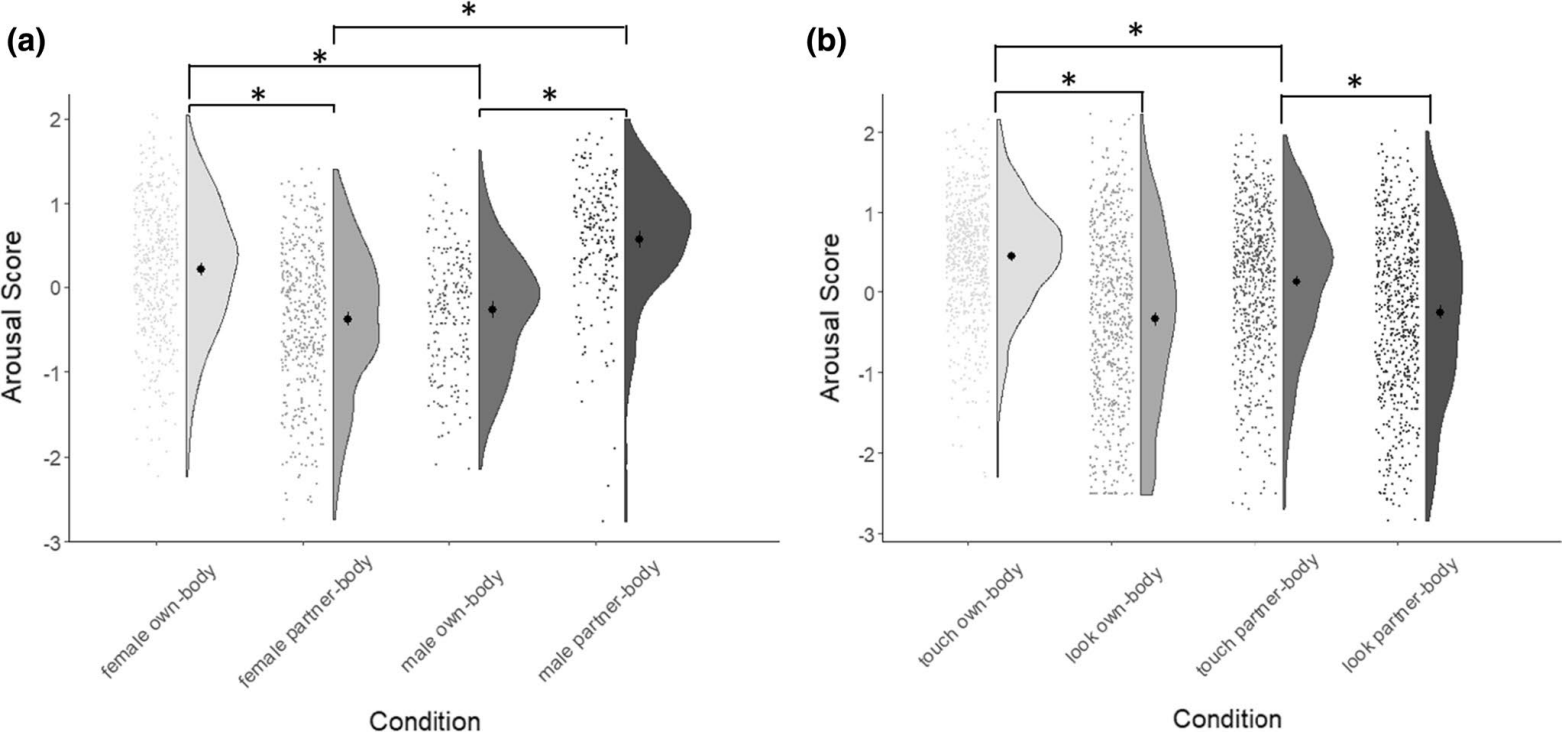
Differential effect of gender (**a**) or modality (**b**) on standardized mean arousal ratings for the sexual body component depending on whether one’s own or a partner’s body is being referred to. Raincloud plots show distribution of individual data points; black central points indicate means, and error bars reflect 95% confidence intervals. Asterisks demonstrate medium or large effect sizes. *N* = 613

For sexual areas, there was a significant Gender × Target Body interaction, *F*(1, 611) = 621.3, *p *< .0001, *η*_p_^2^ = .504. Men gave higher arousal ratings to the sexual body parts of a partner than they did to those same parts on their own body, *M*_Own_ = − 0.27, 95% CI = [− 0.37, − 0.17], *M*_Partner_ = 0.57, 95% CI = [0.48, 0.67], *t*(205) = − 18.0, *p *< .0001, *d *= 1.23. Women showed the opposite pattern; they rated sexual areas as more arousing on their own body than on a partner’s body, *M*_Own_ = 0.22, 95% CI = [0.14, 0.30], *M*_Partner_ = − 0.38, 95% CI = [− 0.46, − 0.29], *t*(406) = 17.8, *p *< .0001, *d *= 0.89. For both target bodies, there were significant gender differences; for their own bodies, women gave significantly higher arousal ratings than men, *t*(611) = 7.47, *p *< .0001, *d *= 0.60; for a partner’s body, they gave significantly lower arousal ratings than men, *t*(611) = − 13.77, *p *< .0001, *d *= 1.11. This is illustrated in Fig. 2 (left panel).

There was also a Modality × Target Body interaction, *F*(1, 611) = 118.9, *p *< .0001, *η*_p_^2^ = .163; there was a general preference toward the tactile modality for both one’s own body, *M*_Touch_ = 0.45, 95% CI = [0.40, 0.51], *M*_Look_ = − 0.34, 95% CI = [− 0.43, − 0.25], *t*(612) = 20.7, *p *< .0001, *d *= .91, and a partner’s body, *M*_Touch_ = 0.14, 95% CI = [0.07, 0.21], *M*_Look_ = − 0.25, 95% CI = [− 0.33, − 0.17], *t*(612) = 16.5, *p* < .0001, *d *= .66, but the tactile preference for own body was significantly larger than for partner-body, *t*(612) = 10.4, *p* < .0001, *d* = 0.44. This is illustrated in Fig. 2 (right panel), using raincloud plots to show the distribution of raw data and summary statistics (produced in R using the procedure from Allen, Poggiali, Whitaker, Marshall, & Kievit, 2018).

**Fig. 3:**
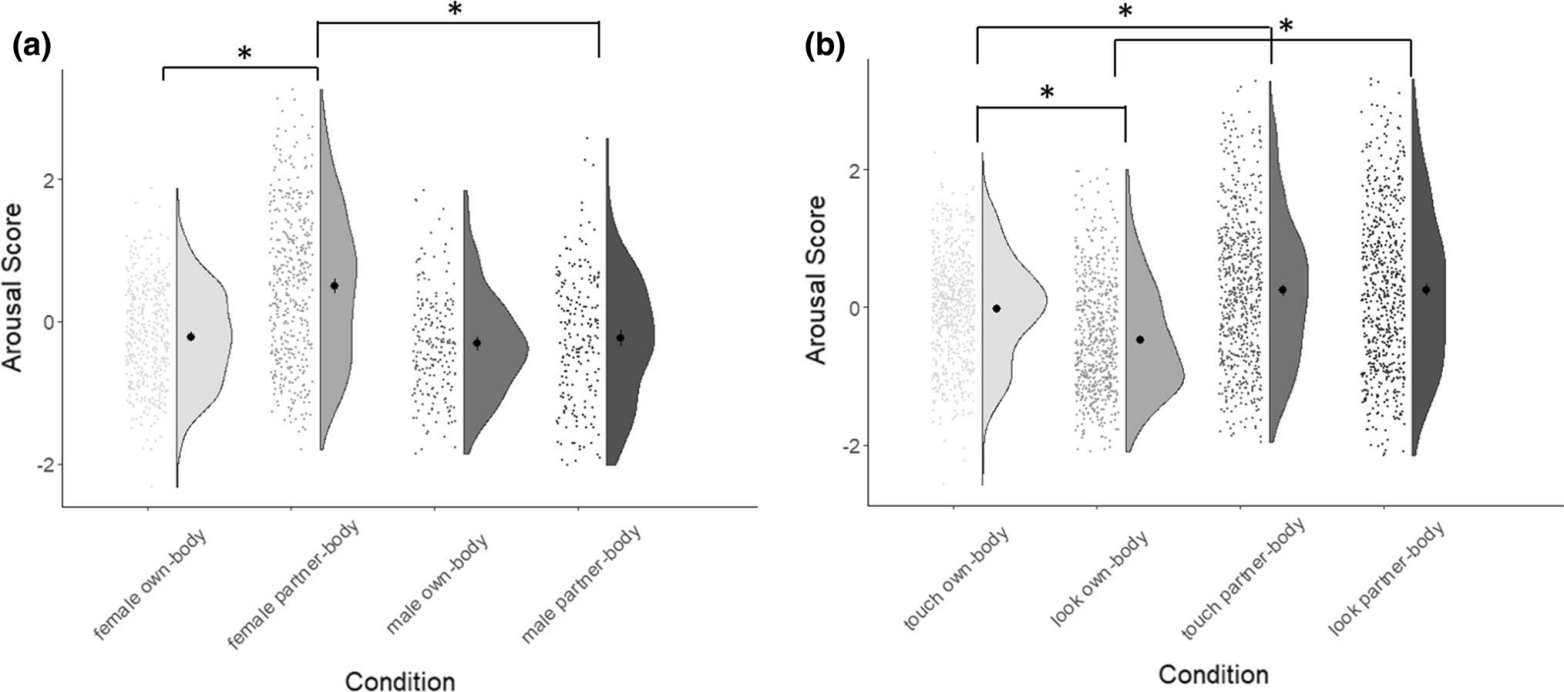
Differential effect of gender (**a**) or modality (**b**) on standardized mean arousal ratings for the sensual body component depending on whether one’s own or a partner’s body is being referred to. Raincloud plots show distribution of individual data points; black central points indicate means, and error bars reflect 95% confidence intervals. Asterisks demonstrate medium or large effect sizes. *N* = 613

For sensual areas, there was again a significant Target Body × Gender interaction, *F*(1, 611) = 111.5, *p* < .0001, *η*_p_^2^ = .154, but the pattern was strikingly different to that shown by the sexual component. Women gave much higher arousal ratings to sensual areas of their partner’s body than their own body, *M*_Own_ = − 0.22, 95% CI = [− 0.28, − 0.15], *M*_Partner_ = 0.49 95% CI = [0.39, 0.59], *t*(406) = − 19.0, *p* < .0001, *d* = 1.07. In contrast, men showed no substantial differences in arousal for their own and a partner’s sensual areas, *M*_Own_ = − 0.31 95% CI = [− 0.41, − 0.21], *M*_Partner_ = − 0.23, 95% CI = [− 0.35, − 0.11], *t*(205) = − 2.0, *p* = .044, *d* = 0.15. Women rated their partner’s sensual areas as significantly more arousing than did men, *t* (611) = 8.43, *p* < .0001, *d *= 0.68. This pattern is illustrated in Fig. 3 (left panel).

There was also a Target Body × Modality interaction revealed for the sensual component, *F*(1, 611) = 147.5, *p* < .0001, *η*_p_^2^ = .194. As with the sexual component, here, touching yielded significantly greater arousal ratings than did looking, for one’s own body *M*_Touch_ = − 0.02, 95% CI = [− 0.08, 0.04], *M*_Look_ = − 0.47, 95% CI = [− 0.54, − 0.41], *d* = 0.71. However, for a partner’s body, there were no modality differences, *M*_Touch_ = 0.24, 95% CI = [0.16, 0.33], *M*_Look_ = 0.25, 95% CI = [0.16, 0.34], *d* = 0.02, suggesting that both touching the partner’s sexual body-parts and looking at them were equally arousing. These results are illustrated in Fig. 3 (right panel).

The following two analyses aimed to test whether there were statistical correspondences between the topographic distribution of erogenous zones on one’s own versus a partner’s body and the topographic distribution of erogenous zones in the tactile versus visual modality.

### The “Erogenous Mirror”: The Correlation Between Individuals’ Maps for Their Own versus a Partner’s Body

This analysis investigated whether the map of sexual pleasure across one’s own body matched the map of sexual pleasure across one’s partner’s body. Specifically, we investigated whether individual differences in ratings for our own body parts, i.e., individual idiosyncratic preferences over and above the sample average, could predict those same idiosyncratic preferences for one’s partner’s body parts. In other words, would an individual who has an above-average preference for being touched, e.g., on their toes, also have an above-average preference for touching their partner’s toes?

To investigate this, individual arousal ratings for each of the 41 body parts were de-meaned by subtracting the group average for that body part for their gender. This gave us a score reflecting how each participant’s ratings deviated from the gender-group “norm.” Then, these de-meaned scores were standardized across each participant, providing scores reflecting within-subject idiosyncratic preferences, uncontaminated by general group consensus or differences in overall whole-body arousal levels.

We then investigated whether there were correlations between these idiosyncratic preferences between one’s own body and a partner’s body. Interestingly, for both the touch and look modalities, the group mean correlation coefficients between individual preferences for giving and receiving were significantly greater than zero; for touch, mean *r*(39) = .33, 95% CI = [0.31, 0.35], *t*(612) = 36.7, *p* < .001, and for look, mean *r*(39) = .30, 95% CI = [0.29, 0.33], *t*(612) = 28.5, *p* < .001. The correlation coefficients for touch and look modalities did not substantially differ, as indicated by a pairwise *t* test which only revealed a small effect, *t*(612) = 2.10, *d* = 0.11. This means that individual preferences for certain body parts, over and above general group consensus, were common both to what we experience on our own body and what we experience on the body of our partner, independently of the sensory modality.

To investigate any gender differences in this “erogenous mirroring,” these scores were then entered into a mixed ANOVA, with modality as a repeated measure and gender as a between-subjects factor. This did not reveal any medium or large effects, suggesting self-other correspondence was equivalent for both men and women.

### Multimodal Erogenous Zones: The Correlation Between Individuals’ Maps for Touching versus Looking

Using the same individual arousal ratings, as calculated for the previous analysis, we then calculated the correlation for each individual between idiosyncratic arousal preferences for touching and looking modalities. The group mean of these correlation coefficients between looking and touching modalities was significantly greater than zero both for one’s own body, mean *r*(39) = .35, 95% CI = [0.33, 0.37], *t*(612) = 35.4, *p* < .0001, *d* = 1.46, and a partner’s body, mean *r*(39) = .56, 95% CI = [0.54, 0.58], *t*(612) = 65.4, *p* < .0001, *d* = 2.67. At the group level, the correlation between visual and tactile modalities was significantly stronger for the partner’s body than for one’s own body, *t*(612) = 18.93, *p* < .0001, *d* = .77. This means that individual preferences for certain body parts, over and above general group consensus, were common both to the tactile and visual modalities, but that this commonality was more marked for one’s ratings of a partner’s body rather than one’s own. As before, gender differences were assessed in a mixed ANOVA with target body as a within-subjects factor, and gender as a between-subjects factor. No main effect or interaction involving gender was present.

### The Mutual Pleasure Index: The Correlation Between Individuals’ Specific Partner-Body Maps and the Mean Maps of Partner’s Gender

**Fig. 4:**
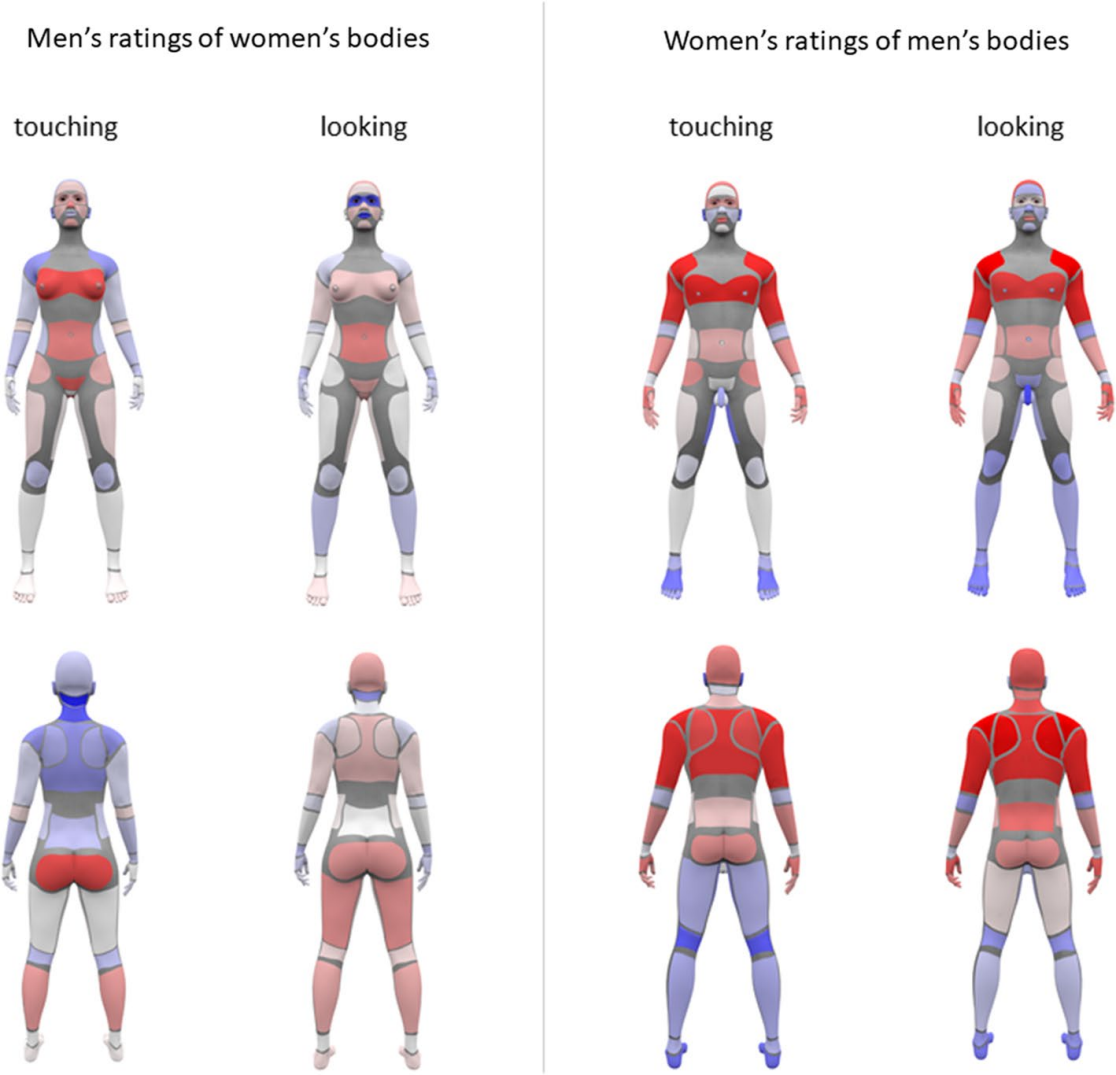
Heat maps showing the distribution of the Mutual Pleasure Index for both touch and look modalities, for men and women separately. Colors represent the residuals from the correlations used to calculate the final mutual pleasure score for each individual. Positive residual scores (the red end of the spectrum) indicate that respondents had a higher preference for touching/looking at that area on the opposite-gender’s body than that gender had for receiving a touch/look on that same part of their own body. Negative residual scores (the blue end of the spectrum) indicate respondents had a lower preference for touching/looking at that area on the opposite-gender’s body than that gender had for receiving a touch/look on that same part of their own body. Residual scores close to zero (white colors) indicate close agreement between respondents’ preferences for an opposite-gender partner’s body and that gender’s preferences for their own body (thus, a high mutual pleasure score) (Color figure online)

Finally, we took data from the heterosexual participants only to investigate whether the individual-level “partner-body” preferences of one gender corresponded to the group-level “own-body” preferences of the opposite gender. In other words, did men’s preferences for touching women correlate with where women liked to be touched, and vice versa? To investigate this, we calculated the correlation between each man’s ratings for a female partner’s body parts with the group average of the females’ ratings of their own body parts. We also did the converse, calculating the correlation between each woman’s ratings for a male partners’ body parts with the group average of the males’ ratings of their own body parts. These calculations yielded “Mutual Pleasure” scores, indicating the extent to which each individual’s preferences for the opposite gender’s body corresponded with the opposite gender’s preferences for their own body. This was done for both the touch and look modalities separately. A heat map illustrating the distribution of mutual pleasure scores across the body surface can be found in Fig. 4.

We then entered these correlation coefficients (one score per individual per condition, calculated across body parts) into a mixed ANOVA with modality as a within-subjects factor and gender as a between-subjects factor. Interestingly, this revealed a medium-sized main effect of gender, *F*(1, 559) = 45.57, *η*_p_^2^ = .075. Men had significantly higher mutual pleasure scores than women, *M*_Men_ = 0.69, 95% CI = [0.67, 0.72], *M*_Women_ = 0.59, 95% CI = [0.57, 0.60], *t*(559) = 6.75, *d* = .60 (medium-sized effect). Therefore, men’s preferences for touching or looking at women aligned more closely with women’s own preferences for being touched or looked at than did the converse. The main effect of modality and the Modality × Gender interaction were not sufficiently large effect sizes to be considered important (*η*_p_^2^ = .05 and .03, respectively).

To investigate the effects of any individual difference variables on the Mutual Pleasure Index, scores were averaged across modalities and entered as the dependent variable into a multiple linear regression, carried out separately for men and women. For men, relationship status, age, sexual satisfaction score, and sensuality score were entered as independent predictors. For women, an additional variable coding whether they were taking the contraceptive pill was also included. As this analysis was relatively exploratory, variables were entered using a stepwise entry method. For women, a significant model was identified containing relationship status, satisfaction score, and pill-status as significant, positive and independent predictors of mutual pleasure score. Women in a relationship had higher mutual pleasure scores than those who were single (*β* = 0.06, 95% CI = [0.02, 0.10], *t* = 3.05, *p* = .002). Women taking the contraceptive pill had higher mutual pleasure scores than those who were not (*β* = 0.05, 95% CI = [0.02, 0.09], *t* = 2.91, *p* = .004), and finally, those who rated themselves as more sexually satisfied had higher mutual pleasure scores (*β* = 0.04, 95% CI = [0.02, 0.07], *t* = 3.44, *p* = .001). Overall, the model was significant, *r* = .31, *F*(3, 326) = 11.24, *p* < .001, but explained a relatively small proportion of the variance (*r*^2^ = .09, 9% variance explained). For men, no suitable model was identified.

To investigate the existence of a mutual pleasure correspondence in non-heterosexual participants with their own gender, we repeated the first analysis just on those who responded with regard to a same-sex partner (including both homosexuals and bi/pansexuals; total *N* = 46; 18 women). First, the same ANOVA was run as in the previous analysis, with modality and gender as factors. This did not reveal any significant effects for modality, *F*(1, 44) = 1.11, *p* = .298, *η*_p_^2^ = .03, nor gender, *F*(1, 44) = 0.44, *p* = .510, *η*_p_^2^ = .01, nor the interaction, *F*(1, 44) = 0.70, *p* = .408, *η*_p_^2^ = 0.02. When comparing the mean mutual pleasure score, averaged across touch and vision modalities, between individuals who gave same-sex responses versus heterosexual participants who gave opposite-sex responses, no significant difference was observed, *M*_Same_ = 0.65, 95% CI = [.62, .69], *M*_Opposite_ = 0.62, 95% CI = [.61, .64], *t* (605) = 1.25, *p* = .213, *d* = 0.19.

## Discussion

Prior knowledge regarding erogenous zones has been restricted to the effects of tactile stimulation of one’s own body. Here, we systematically compared the intensity and distribution of erogenous zones mapped both on one’s own body and on a partner’s body, in response to both tactile and visual stimulation.

As expected, participants gave high ratings to “sexual” areas such as the genitals, and also other areas often involved in sexual interactions, such as the mouth, nipples, and buttocks. Low ratings were given to areas such as knees, elbows, and the chin. This pattern of ratings was very similar to what has been found in previous studies, which solely focussed on tactile stimulation of one’s own body (Nummenmaa et al., 2016; Turnbull et al., 2014; Younis et al., 2016). The pattern was also consistent with gaze behavior toward nude bodies, where highly rated sexual areas are fixated earlier and longer and are associated with elevated physiological arousal (e.g., Nummenmaa, Hietanen, Santtila, & Hyönä, 2012). From the erogenous ratings, we identified three main groups of body areas which elicited similar ratings of erogeneity across participants and contexts, comprising of “sexual,” “sensual,” and “non-sexual” areas. The three extracted components broadly agree with the findings of Turnbull et al. (2014) who also extracted three components from erogenous ratings of body parts. However, in their data, the “erogenous” component included both highly sexual areas (e.g., the genitals) and more sensual areas (e.g., the nape of the neck), whereas our data allowed us to separate these into two distinct components.

Although we observed generally very high agreement between men and women across our sample, gender strongly modulated the effects of target body for both the sexual and sensual body-part components. Men rated sexual areas on their partner’s body as significantly more arousing than the same parts on their own body. In contrast, women rated sexual areas on their *own* body as significantly more arousing than those areas of their partner’s body. This result was consistent with a number of studies that have investigated gender differences in visual attention to erotic stimuli (Rupp & Wallen, 2008). For example, Lykins et al. (2008) found that men looked at opposite-sex figures significantly longer than did women, whereas women looked at same-sex figures significantly longer than did men. This result may also be linked to the importance to women of being perceived as sexually desirable in another’s eyes (Bogaert & Brotto, 2014).

A different pattern of results was observed for the sensual areas of the body. Here, women rated their partner’s sensual areas as significantly more arousing than their own sensual areas. There were no meaningful differences in men’s ratings. Men’s higher ratings of arousal for their partner’s sexual areas over their own were consistent with the finding that men are more easily aroused by visual erotica than are women (Hamann, Herman, Nolan, & Wallen, 2004; Herz & Cahill, 1997). However, our data did not show any specific modulation of the gender effect by modality. Men’s apparent visual preference (Rupp & Wallen, 2008) may in some cases be more accurately defined as a preference toward the partner’s body over their own, in both tactile and visual modalities. For the sensual areas, women showed a significant, stronger preference toward their partner’s sensual body parts rather than their own, independently of modality. Taken together, these gender differences suggest that in sexuality men are more focussed on the partner than women are, but in sensuality, women are more focussed on the partner than men are, a difference that may also relate to a greater preference for foreplay by women versus men (Miller & Byers, 2004). This is also consistent with findings that women visually fixate less on the genital region when viewing sexual pictures of the opposite sex, and fixate more on the stomach region (an area loading onto the “sensual” component here), than men do (Bolmont et al., 2017).

Not surprisingly, both the sensual and sexual areas of one’s own body showed an effect of modality. Participants reported significantly higher arousal when they were touched than when they were looked at. For a partner’s body, these differences were significantly smaller (for sexual areas) or absent (for sensual areas). These differences may reflect differences in the phenomenology across these conditions. When being touched by another person, the touch fulfills a dual role; in sexual contexts, it functions both to provide pleasurable, active tactile information to the person touching (Gentsch et al., 2015), and also to provide pleasurable passive tactile input to the receiver of the touch. Thus, when touched, one is both an object and subject of the touch. This dual nature is less defined in the visual modality, where being looked at by other person primarily provides the “looker” with pleasurable visual stimulation, while the receiver of the look is the object of the looking act. Therefore, the preference for tactile over visual stimulation in the own-body condition may be reformulated as a preference for being a subject rather than an object, or a preference for reciprocity rather than objectification, as evidence suggests that being looked at and its resulting objectification can be experienced positively or negatively depending on context (Emery, 2000; Meltzer, McNulty, & Maner, 2017).

When rating the erogeneity of a partner’s body, there was not such a strong differentiation between touching and looking. Two considerations are relevant here. First, when we consider the experience and function of active touch and visual attention to a partner’s body, for both modalities, the questionnaire respondent is the subject of the stimulation, rather than the object. Second, visual and tactile sensory information is more readily integrated in the partner-body condition over the own-body condition, because they emanate directly from the active exploration of the same spatial source (i.e., the partner’s body) and so both can contribute to the formation of a multimodal partner-body representation. This multimodal representation of the partner’s body may serve an erotic function in itself and may also play an important role in the vicarious pleasure potentially derived from a somatosensory mirror mechanism when touching one’s partner (Keysers & Gazzola, 2009). In contrast, in the own-body condition, visual experience is more indirectly linked to the body, as it involves a second-order inference involving the observation of the partner observing one’s body.

Despite these modality differences in the overall intensity of arousal on a group level, there was generally a strong correlation between individuals’ self-reported arousal ratings for tactile and visual modalities across body parts. This suggests that the topographic distribution of arousal across the body was similar for imagined visual and tactile stimulation; in other words, if an individual found tactile stimulation of a certain body part arousing, either on their own or their partner’s body, they were more likely to also find visual attention toward that same area arousing. This pattern suggests that perceiving another’s visual attention directed to parts of one’s body may activate some of the same arousal pathways as does being touched in those areas. This may reflect a predictive mechanism. Before touching an object in the environment, an individual regularly directs visual attention toward it, which signals their intentions (Pierno et al., 2006). Thus, in intimate contexts, perceiving one’s partner directing their attention toward a part of your one’s body is likely to elicit an anticipation of receiving touch in that specific area.

Corresponding areas of the somatosensory cortex are activated both when an individual is touched and also during the anticipation of touch (Carlsson, Petrovic, Skare, Petersson, & Ingvar, 2000; Drevets et al., 1995), and the same holds for mental imagery of touch as compared to physical touch (Yoo, Freeman, McCarthy, & Jolesz, 2003). Similarly, imagined genital stimulation activates several of the same brain areas, including genital regions of S1, as actual genital stimulation (Wise, Frangos, & Komisaruk, 2016). Furthermore, visually induced anticipation of touch enhances body-awareness (Ferri, Chiarelli, Merla, Gallese, & Costantini, 2013) and visual enhancement of tactile sensations has been widely reported (Haggard, 2006; Serino, Pizzoferrato, & Làdavas, 2008). Therefore, the presence of such erogenous multimodal correspondence maps, i.e., correspondence between the tactile map (of where an individual likes to be touched) and the visual map (of where the same individual likes to be looked at), may play an important role in anticipation but also amplification of arousal during interpersonal sexual activities. Furthermore, it is interesting to consider what role this could play in individuals who engage in exhibitionism; the desire to be “looked at” nude, or while engaging in sexual acts, suggests these individuals have an enhanced intensity of erogenous zones elicited by visual attention to their body. Future research could investigate the topographic distribution of erogenous maps across modalities in exhibitionists to delve into this topic further.

Importantly, there was also a clear correspondence, over and above group consensus, between the erogenous zone maps referring to one’s own body and one’s experience of a partner’s body. Individuals who idiosyncratically rated certain body parts as arousing on their own body were more likely to find that same body part arousing on their partner’s body. This correspondence was similar for visual and tactile modalities, and no meaningful gender differences were present. This may reflect an association or expectation effect, whereby focus on certain areas of a body (regardless of whether it is one’s own or a partner’s) activates the expectation of sexual interactions. Given that these areas have the same topographical distribution on both one’s own and one’s partner’s body, even once the effects of commonly erogenous zones such as the genitals have been controlled for, there may be a more specific somatotopic interpersonal “erogenous mirror,” whereby touching or looking at an area of a partner’s body may activate the same representation as that activated by being touched or looked at oneself. The existence of such a mirror is plausible, as there are already reported vicariously activated mirror-neuron systems for the interpersonal mapping of tactile, motor and even emotional experiences in non-sexual contexts (for review, see Keysers & Gazzola, 2009). This possibility now needs to be directly tested in future research. One important consideration is how this interpersonal erogenous mirror is affected by the type of interpersonal relationship being studied; for example, committed, long-term romantic partners may have a higher level of mirroring than more casual, one-off sexual encounters. Existing evidence for the top-down modulation of vicarious sensory activation by social factors lends weight to this theory (Avenanti, Sirigu, & Aglioti, 2010; Serino, Giovagnoli, & Làdavas, 2009), which could be an interesting avenue for further study.

Finally, an analysis of a “Mutual Pleasure Index” between genders on heterosexual participants assessed how, on a group level, participants’ arousal maps for the opposite gender’s body corresponded to the opposite gender’s arousal map for their own body. Men’s arousal ratings for women’s bodies aligned more closely with women’s average arousal ratings for their own bodies, rather than the converse. This could be interpreted within an evolutionary psychology framework; it may be possible that males have evolved to derive sexual pleasure from stimulating areas of the female body that are most likely to give the female pleasure, and therefore most likely to allow the male to retain access to the female (Gray, 2013). Although this idea is consistent with the classic theory of males being “ardent,” while females are “choosy,” (Bateman, 1948), further investigation is required to explore these possibilities. Interestingly, non-heterosexual individuals who answered about a same-sex partner did not show any significant differences in the extent of their mutual pleasure scores, as compared to heterosexual individuals, indicated that there were no differences in the extent to which the body areas that same-sex attracted individuals enjoyed to touch/look at on a same-sex partner’s body corresponded to where those partners enjoyed being touched/looked at on their own bodies.

Importantly, for the heterosexual participants, only the women’s mutual pleasure scores were predicted by demographic variables. Higher scores, indicating higher gender-group correspondence, were independently associated with being in a relationship rather than being single, having higher self-reported sexual satisfaction, and being on the contraceptive pill. The finding that women who were in relationships, and more sexually satisfied, had higher mutual pleasure scores could be interpreted in two different ways, depending on the assumed causal direction. For example, some women may be in current sexual relationships and experience high sexual satisfaction, *because* their sexual desires toward men happen to align more closely with men’s desires for their own bodies. This could result in them not only being more sexually satisfied, but also being highly desirable sexual partners, as they are able to simultaneously achieve personal sexual satisfaction while satisfying their partner. Alternatively, close gender-group correspondence may instead be an *effect* of sexual satisfaction and of being in a relationship; women’s personal sexual preferences may gradually change to more closely match what they learn their male partner enjoys. The correlational nature of the current data does not allow us to disentangle these interpretations.

The effect of the contraceptive pill on mutual pleasure scores was also particularly interesting and highlights a potential biologically mediated mechanism which requires further investigation. Again, an evolutionary account may be used to explain this finding. Women on the contraceptive pill, whose effects mimic some of the hormonal changes found in pregnancy, are thought to be more motivated toward long-term pair bonding and mate retention, than those who are hormonally fertile (Welling, Puts, Roberts, Little, & Burriss, 2012). Therefore, our finding that women on the pill had a greater congruency between what they reportedly enjoyed doing to their male partner and what males enjoyed being done to them may have been linked to a greater motivation for mate retention. In contrast, normal-cycling females who are in estrus have an increased desire for orgasm, increased sexual fantasy (Regan, 1996), increased desire for extra-pair copulation (Grebe, Emery Thompson, & Gangestad, 2016), and are choosier with regard to their sexual partners (Haselton & Gangestad, 2006) than hormonally non-fertile females. This, arguably more short-term evolutionary drive, may have been linked to a reduced need for congruence between women’s sexual desires and those of the male.

The study had a number of key limitations that are important to note. First, as we were measuring self-reported experiences, one limitation was that participants may have not recalled their behaviors accurately, and this is something that we were unable to control for. Furthermore, we did not record additional details regarding exactly what experiences participants were rating when they were answering about a sexual partner; for example, participants could have been ratings experiences from a current relationship, or a past relationship, and this may have affected the intensity of the ratings. Another limitation was due to the fact that our sample was self-selected, and the study was advertised primarily across platforms linked with academic institutions and social media, it was unlikely to reflect the true diversity of the population. However, in comparison with laboratory-based studies, internet samples have been found to be relatively diverse with respect to participant demographics, and although they are subject to high attrition and noisy data, the sample sizes achieved can be orders of magnitude larger, increasing power and reliability of results (Gosling, Vazire, Srivastava, & John, 2004). In addition, the anonymous nature of the questionnaire may have allowed participants to be more honest with regard to their sexual experiences, lessening social desirability biases that could have been present in a laboratory setting (Ramo, Hall, & Prochaska, 2011).

Even though our results derived from self-reported arousal as measured using a questionnaire, the use of actual tactile or visual stimulation and the presence of a real sexual partner would provide a more direct and ecological assessment of our findings in future studies. Nonetheless, our multimodal and interpersonal approach highlights hitherto unexplored aspects of the erogeneity of our bodies. Previous studies have only focussed on erogenous experience elicited by tactile stimulation of various body parts; therefore, the distribution of erogenous zones stimulated by vision was hitherto unknown. Furthermore, no study to date had investigated one’s own erogenous experience when stimulating a partner’s body, despite this being an important aspect of sexual interactions. In the current study, we found substantial gender differences in how strongly areas of one’s own and one’s partner’s body can elicit arousal, as well as differences in arousal intensity elicited by visual versus tactile stimulation. Despite these differences in arousal intensity, visual attention to specific body parts appears to result in similarly distributed topographic patterns of arousal to those elicited by tactile stimulation. Furthermore, we found clear correspondence between individuals’ topographic distributions of erogenous zones mapped onto their own and their partners’ bodies, across the sample. This suggests that the erogeneity of body parts may be represented on a somatotopic map that can be activated similarly for both self and other-related stimuli. Further investigation into the neural basis for these self-other and visual-tactile correspondences in erogenous zone distribution could reveal further insights into their basis and function during sexual interactions.

## Electronic supplementary material

Below is the link to the electronic supplementary material.

Supplementary material 1 (DOCX 37 kb)
