# Supplementary material for: The Erogenous Mirror: Intersubjective and Multisensory Maps of Sexual Arousal in Men and Women
Source: Arch Sex Behav. 2020 Jun 12;49(8):2919–33. doi: 10.1007/s10508-020-01756-1 (PMC7641941; doi:10.1007/s10508-020-01756-1)
Supplement: Supplementary file 1 — Supplementary material 1 (DOCX 37 kb) [file 10508_2020_1756_MOESM1_ESM.docx]

**Supplemental Information**

*Instructions to participants*

“This questionnaire aims to investigate the parts of the body which give sexual pleasure. We aim to investigate which areas of the body are able to arouse sexual feelings, when they are touched or looked at. We also want to find out which areas of a partner’s body you find arousing when you look at them or touch them.”

“You will now see an “Arousal Scale” which consists of 41 body areas which you are to rate as arousing or not. You are to rate the level of arousal of the body area on an 11-point scale, where 1 is not at all arousing, and 11 is extremely arousing.”

“Please rate the strength of your sexual arousal (or rate how much you are sexually aroused) when [your partner touches you on your][your partner looks at your][you touch your partner’s][you look at your partner’s] different body parts. Please fill this in with reference to your current or past sexual partner(s). If you have noted differences between partners, rate your most intense experiences.”

To ask participants about their sexual orientation, they were asked to “Please select your sexual orientation from the list below”, with the options provided in a drop down menu consisting of Heterosexual, Bisexual/Pansexual, Homosexual, Asexual, Other(free entry) and ‘Prefer not to say’.

*S2. Descriptive Data*

*1. Mean responses to each body part, for each condition, split by gender*

*Own-body/Touch*

|  | Women | |  | Men | |
| --- | --- | --- | --- | --- | --- |
| Body part | *M* | *SD* |  | *M* | *SD* |
| Forehead | 3.20 | 2.27 |  | 3.60 | 2.35 |
| Eye and temple | 3.68 | 2.61 |  | 4.15 | 2.54 |
| Ears | 6.22 | 3.17 |  | 6.43 | 2.87 |
| Nose | 2.57 | 1.88 |  | 3.33 | 2.41 |
| Cheeks | 4.35 | 2.59 |  | 4.68 | 2.56 |
| Mouth and Lips | 8.80 | 2.17 |  | 8.26 | 2.29 |
| Chin | 3.07 | 2.35 |  | 3.57 | 2.38 |
| Nape of Neck | 8.06 | 2.58 |  | 6.89 | 2.60 |
| Shoulders | 5.30 | 2.76 |  | 4.79 | 2.49 |
| Upper Arm | 4.19 | 2.47 |  | 4.30 | 2.43 |
| Elbows | 2.46 | 1.99 |  | 2.87 | 2.15 |
| Forearm | 4.06 | 2.44 |  | 4.07 | 2.33 |
| Wrists | 4.14 | 2.71 |  | 3.83 | 2.58 |
| Hands | 5.00 | 2.75 |  | 5.08 | 2.63 |
| Fingers | 4.83 | 2.91 |  | 4.92 | 2.76 |
| Breasts/chest | 8.80 | 2.21 |  | 6.46 | 2.61 |
| Nipples | 9.00 | 2.39 |  | 6.62 | 3.14 |
| Stomach | 5.32 | 2.81 |  | 5.69 | 2.68 |
| Sides | 6.03 | 2.81 |  | 5.39 | 2.62 |
| Bellybutton | 4.04 | 2.93 |  | 4.10 | 2.78 |
| Pubic hairline | 7.50 | 2.73 |  | 7.65 | 2.61 |
| Hips | 6.85 | 2.59 |  | 5.60 | 2.70 |
| Vagina/scrotum | 9.95 | 1.81 |  | 8.53 | 2.91 |
| Clitoris/penis | 10.36 | 1.69 |  | 10.38 | 1.58 |
| Perineum | 6.85 | 3.31 |  | 8.07 | 2.92 |
| Inner thighs | 8.58 | 2.23 |  | 7.99 | 2.36 |
| Outer thighs | 5.87 | 2.64 |  | 5.09 | 2.46 |
| Knee Caps | 2.98 | 2.31 |  | 2.83 | 2.02 |
| Shin | 2.85 | 2.21 |  | 2.76 | 1.99 |
| Ankles | 3.19 | 2.48 |  | 2.99 | 2.22 |
| Foot | 3.52 | 2.66 |  | 3.65 | 2.69 |
| Toes | 3.18 | 2.62 |  | 3.26 | 2.59 |
| Head and Hair | 6.88 | 3.00 |  | 6.26 | 2.76 |
| Back of Neck | 7.81 | 2.86 |  | 6.62 | 2.87 |
| Shoulder Blades | 5.37 | 2.99 |  | 5.05 | 2.68 |
| Upper Back | 6.06 | 2.89 |  | 5.34 | 2.74 |
| Lower Back | 7.13 | 2.67 |  | 6.12 | 2.75 |
| Buttocks | 7.88 | 2.65 |  | 7.12 | 2.86 |
| Back of thighs | 6.56 | 2.75 |  | 5.77 | 2.76 |
| Behind Knees | 4.23 | 2.88 |  | 4.21 | 2.72 |
| Calves/Back of Shins | 3.50 | 2.54 |  | 3.93 | 2.51 |

*Own-body/Look*

|  | Women | |  | Men | |
| --- | --- | --- | --- | --- | --- |
| Body part | *M* | *SD* |  | *M* | *SD* |
| Forehead | 2.47 | 2.15 |  | 2.90 | 2.43 |
| Eye and temple | 5.65 | 3.47 |  | 5.13 | 3.38 |
| Ears | 2.87 | 2.63 |  | 2.92 | 2.44 |
| Nose | 2.18 | 1.91 |  | 2.74 | 2.23 |
| Cheeks | 3.08 | 2.58 |  | 3.32 | 2.73 |
| Mouth and Lips | 7.38 | 3.16 |  | 6.19 | 3.15 |
| Chin | 2.23 | 1.97 |  | 2.71 | 2.31 |
| Nape of Neck | 4.80 | 3.35 |  | 3.63 | 2.94 |
| Shoulders | 3.51 | 2.77 |  | 3.43 | 2.59 |
| Upper Arm | 2.40 | 2.01 |  | 3.16 | 2.53 |
| Elbows | 1.90 | 1.67 |  | 2.16 | 1.90 |
| Forearm | 2.26 | 1.90 |  | 2.95 | 2.36 |
| Wrists | 2.64 | 2.31 |  | 2.61 | 2.19 |
| Hands | 3.31 | 2.72 |  | 3.37 | 2.64 |
| Fingers | 3.04 | 2.61 |  | 3.05 | 2.53 |
| Breasts/chest | 7.64 | 3.00 |  | 4.80 | 2.97 |
| Nipples | 7.45 | 3.18 |  | 4.96 | 3.11 |
| Stomach | 3.86 | 2.89 |  | 3.74 | 2.73 |
| Sides | 3.98 | 2.94 |  | 3.09 | 2.37 |
| Bellybutton | 3.72 | 2.95 |  | 3.37 | 2.63 |
| Pubic hairline | 6.00 | 3.37 |  | 6.01 | 3.31 |
| Hips | 5.54 | 3.28 |  | 4.00 | 2.87 |
| Vagina/scrotum | 7.48 | 3.30 |  | 6.36 | 3.57 |
| Clitoris/penis | 7.47 | 3.32 |  | 8.38 | 3.06 |
| Perineum | 5.25 | 3.63 |  | 5.59 | 3.64 |
| Inner thighs | 5.89 | 3.28 |  | 5.01 | 3.16 |
| Outer thighs | 4.22 | 3.09 |  | 3.37 | 2.60 |
| Knee Caps | 2.00 | 1.76 |  | 2.19 | 1.86 |
| Shin | 2.15 | 1.93 |  | 2.17 | 1.80 |
| Ankles | 2.18 | 1.93 |  | 2.23 | 2.00 |
| Foot | 2.26 | 2.02 |  | 2.36 | 2.15 |
| Toes | 2.06 | 1.87 |  | 2.36 | 2.23 |
| Head and Hair | 4.26 | 3.24 |  | 3.90 | 3.03 |
| Back of Neck | 4.45 | 3.46 |  | 3.49 | 2.86 |
| Shoulder Blades | 3.43 | 2.92 |  | 3.38 | 2.74 |
| Upper Back | 3.88 | 3.14 |  | 3.77 | 2.81 |
| Lower Back | 4.80 | 3.34 |  | 3.83 | 2.90 |
| Buttocks | 6.62 | 3.40 |  | 5.47 | 3.38 |
| Back of thighs | 4.14 | 3.16 |  | 3.60 | 2.88 |
| Behind Knees | 2.30 | 2.21 |  | 2.49 | 2.23 |
| Calves/Back of Shins | 2.46 | 2.28 |  | 2.72 | 2.36 |

*Partner-body/Touch*

|  | Women | |  | Men | |
| --- | --- | --- | --- | --- | --- |
| Body part | *M* | *SD* |  | *M* | *SD* |
| Forehead | 3.23 | 2.65 |  | 3.98 | 2.62 |
| Eye and temple | 4.22 | 3.17 |  | 4.56 | 2.81 |
| Ears | 4.84 | 3.23 |  | 5.62 | 3.01 |
| Nose | 2.70 | 2.40 |  | 3.84 | 2.52 |
| Cheeks | 3.95 | 2.95 |  | 5.17 | 2.73 |
| Mouth and Lips | 8.61 | 2.47 |  | 8.41 | 2.44 |
| Chin | 3.27 | 2.69 |  | 4.13 | 2.75 |
| Nape of Neck | 6.22 | 3.07 |  | 6.42 | 2.82 |
| Shoulders | 5.86 | 3.28 |  | 5.08 | 2.83 |
| Upper Arm | 5.27 | 3.25 |  | 4.59 | 2.81 |
| Elbows | 2.22 | 1.95 |  | 3.26 | 2.36 |
| Forearm | 4.36 | 3.11 |  | 4.30 | 2.62 |
| Wrists | 3.42 | 2.77 |  | 4.23 | 2.85 |
| Hands | 5.47 | 3.10 |  | 5.37 | 2.76 |
| Fingers | 4.96 | 3.24 |  | 5.12 | 2.90 |
| Breasts/chest | 7.57 | 2.75 |  | 9.76 | 1.70 |
| Nipples | 5.95 | 3.25 |  | 9.58 | 1.85 |
| Stomach | 5.65 | 3.00 |  | 6.54 | 2.70 |
| Sides | 5.15 | 3.06 |  | 5.93 | 2.77 |
| Bellybutton | 3.75 | 2.97 |  | 5.75 | 2.95 |
| Pubic hairline | 7.10 | 3.17 |  | 8.55 | 2.50 |
| Hips | 5.89 | 3.20 |  | 7.29 | 2.49 |
| Vagina/scrotum | 7.47 | 3.20 |  | 10.03 | 2.00 |
| Clitoris/penis | 9.73 | 2.07 |  | 10.10 | 1.82 |
| Perineum | 6.05 | 3.65 |  | 8.72 | 2.71 |
| Inner thighs | 6.59 | 3.06 |  | 8.57 | 2.20 |
| Outer thighs | 4.79 | 2.97 |  | 6.27 | 2.67 |
| Knee Caps | 2.40 | 2.13 |  | 3.21 | 2.45 |
| Shin | 2.45 | 2.18 |  | 3.38 | 2.38 |
| Ankles | 2.24 | 1.97 |  | 3.69 | 2.65 |
| Foot | 2.26 | 2.07 |  | 4.07 | 3.03 |
| Toes | 2.27 | 2.16 |  | 3.87 | 3.02 |
| Head and Hair | 6.50 | 3.27 |  | 6.64 | 2.99 |
| Back of Neck | 6.44 | 3.22 |  | 6.65 | 2.90 |
| Shoulder Blades | 5.80 | 3.27 |  | 5.28 | 2.85 |
| Upper Back | 6.27 | 3.24 |  | 5.67 | 2.87 |
| Lower Back | 6.02 | 3.18 |  | 6.93 | 2.74 |
| Buttocks | 7.33 | 3.10 |  | 9.13 | 2.38 |
| Back of thighs | 4.90 | 3.20 |  | 6.71 | 2.85 |
| Behind Knees | 2.64 | 2.28 |  | 4.21 | 2.94 |
| Calves/Back of Shins | 3.01 | 2.63 |  | 4.68 | 2.95 |

*Partner-body/Look*

|  | Women | |  | Men | |
| --- | --- | --- | --- | --- | --- |
| Body part | *M* | *SD* |  | *M* | *SD* |
| Forehead | 3.11 | 2.66 |  | 3.80 | 2.77 |
| Eye and temple | 6.10 | 3.53 |  | 5.91 | 3.31 |
| Ears | 3.26 | 2.71 |  | 4.03 | 2.88 |
| Nose | 2.81 | 2.54 |  | 3.71 | 2.88 |
| Cheeks | 3.59 | 2.90 |  | 4.64 | 2.89 |
| Mouth and Lips | 7.81 | 2.77 |  | 7.59 | 2.97 |
| Chin | 3.03 | 2.69 |  | 3.74 | 2.62 |
| Nape of Neck | 5.23 | 3.26 |  | 5.44 | 3.04 |
| Shoulders | 5.99 | 3.37 |  | 4.80 | 3.03 |
| Upper Arm | 5.27 | 3.33 |  | 4.11 | 2.77 |
| Elbows | 2.16 | 1.88 |  | 2.93 | 2.39 |
| Forearm | 4.16 | 3.11 |  | 3.72 | 2.67 |
| Wrists | 3.11 | 2.76 |  | 3.51 | 2.62 |
| Hands | 5.20 | 3.31 |  | 4.39 | 2.99 |
| Fingers | 4.51 | 3.33 |  | 4.23 | 2.96 |
| Breasts/chest | 7.02 | 3.07 |  | 9.40 | 2.23 |
| Nipples | 5.42 | 3.27 |  | 9.08 | 2.51 |
| Stomach | 5.04 | 3.17 |  | 6.08 | 2.83 |
| Sides | 4.33 | 3.05 |  | 5.30 | 2.91 |
| Bellybutton | 3.59 | 2.96 |  | 5.34 | 3.03 |
| Pubic hairline | 6.44 | 3.31 |  | 7.95 | 2.90 |
| Hips | 5.10 | 3.31 |  | 6.73 | 2.96 |
| Vagina/scrotum | 6.06 | 3.45 |  | 9.22 | 2.59 |
| Clitoris/penis | 8.90 | 2.70 |  | 9.36 | 2.44 |
| Perineum | 4.86 | 3.51 |  | 7.97 | 3.12 |
| Inner thighs | 5.42 | 3.26 |  | 7.42 | 2.85 |
| Outer thighs | 4.16 | 3.00 |  | 5.50 | 3.07 |
| Knee Caps | 2.24 | 2.03 |  | 2.90 | 2.31 |
| Shin | 2.32 | 2.10 |  | 3.03 | 2.30 |
| Ankles | 2.18 | 1.95 |  | 3.23 | 2.54 |
| Foot | 2.09 | 1.84 |  | 3.65 | 2.95 |
| Toes | 2.02 | 1.82 |  | 3.38 | 2.77 |
| Head and Hair | 5.67 | 3.47 |  | 5.99 | 3.25 |
| Back of Neck | 5.32 | 3.39 |  | 5.69 | 3.27 |
| Shoulder Blades | 5.78 | 3.33 |  | 5.03 | 3.12 |
| Upper Back | 5.94 | 3.33 |  | 5.68 | 3.13 |
| Lower Back | 5.58 | 3.24 |  | 6.19 | 3.17 |
| Buttocks | 7.02 | 3.19 |  | 8.70 | 2.86 |
| Back of thighs | 4.47 | 3.27 |  | 6.09 | 3.12 |
| Behind Knees | 2.47 | 2.32 |  | 3.65 | 2.81 |
| Calves/Back of Shins | 3.06 | 2.65 |  | 4.24 | 2.97 |

*2. Body parts ordered by preference for men vs. women*

*Own-body/Touch*

| **WOMEN** | **MEN** |
| --- | --- |
| Elbows | Elbows |
| Nose | Shin |
| Shin | Nose |
| Knee Caps | Knee Caps |
| Chin | Ankles |
| Toes | Toes |
| Ankles | Chin |
| Forehead | Forehead |
| Calves/Back of Shins | Foot |
| Foot | Calves/Back of Shins |
| Eye and temple | Eye and temple |
| Bellybutton | Wrists |
| Forearm | Bellybutton |
| Wrists | Forearm |
| Upper Arm | Behind Knees |
| Behind Knees | Upper Arm |
| Cheeks | Cheeks |
| Fingers | Fingers |
| Hands | Hands |
| Shoulders | Shoulders |
| Stomach | Shoulder Blades |
| Shoulder Blades | Stomach |
| Outer thighs | Outer thighs |
| Sides | Sides |
| Upper Back | Upper Back |
| Ears | Ears |
| Back of thighs | Back of thighs |
| Perineum | Hips |
| Hips | Head and Hair |
| Head and Hair | Lower Back |
| Lower Back | Perineum |
| Pubic hairline | Back of Neck |
| Back of Neck | Pubic hairline |
| Buttocks | Buttocks |
| Nape of Neck | Nape of Neck |
| Inner thighs | Breasts/chest |
| Mouth/Lips | Nipples |
| Breasts/chest | Inner thighs |
| Nipples | Mouth/Lips |
| Vagina/scrotum | Vagina/scrotum |
| Clitoris/penis | Clitoris/penis |

*Own-body/Look*

| **WOMEN** | **MEN** |
| --- | --- |
| Elbows | Elbows |
| Knee Caps | Shin |
| Toes | Knee Caps |
| Shin | Ankles |
| Nose | Foot |
| Ankles | Toes |
| Chin | Behind Knees |
| Foot | Wrists |
| Forearm | Chin |
| Behind Knees | Calves/Back of Shins |
| Upper Arm | Nose |
| Calves/Back of Shins | Forehead |
| Forehead | Ears |
| Wrists | Forearm |
| Ears | Fingers |
| Fingers | Sides |
| Cheeks | Upper Arm |
| Hands | Cheeks |
| Shoulder Blades | Hands |
| Shoulders | Bellybutton |
| Bellybutton | Outer thighs |
| Stomach | Shoulder Blades |
| Upper Back | Shoulders |
| Sides | Back of Neck |
| Back of thighs | Back of thighs |
| Outer thighs | Nape of Neck |
| Head and Hair | Stomach |
| Back of Neck | Upper Back |
| Nape of Neck | Lower Back |
| Lower Back | Head and Hair |
| Perineum | Hips |
| Hips | Breasts/chest |
| Eye and temple | Nipples |
| Inner thighs | Inner thighs |
| Pubic hairline | Eye and temple |
| Buttocks | Buttocks |
| Mouth/Lips | Perineum |
| Nipples | Pubic hairline |
| Clitoris/penis | Mouth/Lips |
| Vagina/scrotum | Vagina/scrotum |
| Breasts/chest | Clitoris/penis |

*Partner-body/Touch*

| **WOMEN** | **MEN** |
| --- | --- |
| Elbows | Knee Caps |
| Ankles | Elbows |
| Foot | Shin |
| Toes | Ankles |
| Knee Caps | Nose |
| Shin | Toes |
| Behind Knees | Forehead |
| Nose | Foot |
| Calves/Back of Shins | Chin |
| Forehead | Behind Knees |
| Chin | Wrists |
| Wrists | Forearm |
| Bellybutton | Eye and temple |
| Cheeks | Upper Arm |
| Eye and temple | Calves/Back of Shins |
| Forearm | Shoulders |
| Outer thighs | Fingers |
| Ears | Cheeks |
| Back of thighs | Shoulder Blades |
| Fingers | Hands |
| Sides | Ears |
| Upper Arm | Upper Back |
| Hands | Bellybutton |
| Stomach | Sides |
| Shoulder Blades | Outer thighs |
| Shoulders | Nape of Neck |
| Hips | Stomach |
| Nipples | Head and Hair |
| Lower Back | Back of Neck |
| Perineum | Back of thighs |
| Nape of Neck | Lower Back |
| Upper Back | Hips |
| Back of Neck | Mouth Lips |
| Head and Hair | Pubic hairline |
| Inner thighs | Inner thighs |
| Pubic hairline | Perineum |
| Buttocks | Buttocks |
| Vagina/Scrotum | Nipples |
| Breasts/chest | Breasts/chest |
| Mouth/Lips | Vagina/Scrotum |
| Clitoris/penis | Clitoris/penis |

*Partner-body/Look*

| **WOMEN** | **MEN** |
| --- | --- |
| Toes | Toes |
| Foot | Knee Caps |
| Elbows | Elbows |
| Ankles | Shin |
| Knee Caps | Ankles |
| Shin | Wrists |
| Behind Knees | Behind Knees |
| Nose | Foot |
| Chin | Nose |
| Calves/Back of Shins | Forearm |
| Forehead | Chin |
| Wrists | Forehead |
| Ears | Ears |
| Bellybutton | Upper Arm |
| Cheeks | Fingers |
| Forearm | Calves/Back of Shins |
| Outer thighs | Hands |
| Sides | Cheeks |
| Back of thighs | Shoulders |
| Fingers | Shoulder Blades |
| Perineum | Sides |
| Stomach | Bellybutton |
| Hips | Nape of Neck |
| Hands | Outer thighs |
| Nape of Neck | Upper Back |
| Upper Arm | Back of Neck |
| Back of Neck | Eye and temple |
| Nipples | Head and Hair |
| Inner thighs | Stomach |
| Lower Back | Back of thighs |
| Head and Hair | Lower Back |
| Shoulder Blades | Hips |
| Upper Back | Inner thighs |
| Shoulders | Mouth/Lips |
| Vagina/Scrotum | Pubic hairline |
| Eye and temple | Perineum |
| Pubic hairline | Buttocks |
| Buttocks | Nipples |
| Breasts/chest | Vagina/Scrotum |
| Mouth/Lips | Clitoris/Penis |
| Clitoris/Penis | Breasts/chest |

Table S3.

Results of Principal Components Analysis showing details of three extracted components

| Component | *Initial Eigenvalues* | | |  | *Loadings* | | |
| --- | --- | --- | --- | --- | --- | --- | --- |
|  | *Total* | *% of Variance* | *Cumulative %* |  | *Total* | *% of Variance* | *Cumulative %* |
| Sensual | 18.89 | 46.08 | 46.08 |  | 8.69 | 21.19 | 21.19 |
| Non-arousing | 3.37 | 8.22 | 54.30 |  | 8.66 | 21.11 | 42.30 |
| Sexual | 2.25 | 5.48 | 59.78 |  | 7.17 | 17.48 | 59.78 |

Table S4.

Principal Components Analysis factor loadings, for the 41 body parts rated.

| Component | *sensual* | *non-arousing* | *sexual* |
| --- | --- | --- | --- |
| Forehead | .48 | **.59** | .11 |
| Eye and temple | **.51** | .31 | .08 |
| Ears | .36 | **.46** | .33 |
| Nose | .40 | **.65** | .11 |
| Cheeks | **.52** | **.53** | .24 |
| Mouth/Lips | **.52** | .12 | **.50** |
| Chin | .49 | **.62** | .11 |
| Nape of Neck | **.55** | .21 | .44 |
| Shoulders | **.79** | .23 | .17 |
| Upper Arm | **.71** | .33 | .10 |
| Elbows | .26 | **.78** | .10 |
| Forearm | **.62** | .47 | .11 |
| Wrists | .47 | **.59** | .18 |
| Hands | **.65** | .43 | .15 |
| Fingers | **.57** | .49 | .18 |
| Breasts/chest | .38 | .07 | **.67** |
| Nipples | .10 | .23 | **.75** |
| Stomach | **.48** | .27 | **.42** |
| Sides | **.47** | .36 | **.44** |
| Bellybutton | .27 | **.50** | .39 |
| Pubic hairline | .21 | .16 | **.69** |
| Hips | .40 | .28 | **.58** |
| Vagina/scrotum | -.03 | .13 | **.80** |
| Clitoris/penis | .16 | .02 | **.69** |
| Perineum | -.04 | .32 | **.67** |
| Inner thighs | .25 | .20 | **.73** |
| Outer thighs | .42 | .37 | **.51** |
| Knee Caps | .25 | **.77** | .16 |
| Shin | .24 | **.74** | .17 |
| Ankles | .17 | **.78** | .21 |
| Foot | .14 | **.76** | .24 |
| Toes | .10 | **.77** | .21 |
| Head and Hair | **.69** | .24 | .33 |
| Back of Neck | **.62** | .26 | .41 |
| Shoulder Blades | **.78** | .23 | .22 |
| Upper Back | **.79** | .21 | .26 |
| Lower Back | **.60** | .23 | .50 |
| Buttocks | .35 | .12 | **.67** |
| Back of thighs | .41 | .37 | **.55** |
| Behind Knees | .26 | .68 | .26 |
| Calves/Back of Shins | .37 | .63 | .24 |

Note. Body areas with loadings > 0.40 are indicated in bold for ease of interpretation. For body parts with loadings > 0.40 on more than one component, the highest loading only is in bold if the difference between their respective coefficients is greater than 0.10. For relatively equal loadings on two components (a difference of less than 0.10), both coefficients are in bold for inspection.

Table S5

*ANOVA results for Mutual Pleasure Index*

| Predictor | *df_Num_* | *df_Den_* | *SS_Num_* | *SS_Den_* | *F* | *p* | η^2^_p_ |
| --- | --- | --- | --- | --- | --- | --- | --- |
| (Intercept) | 1 | 603 | 135.71 | 36.06 | 2269.48 | .000 | .79 |
| Gender | 1 | 603 | 0.13 | 36.06 | 2.20 | .138 | .00 |
| Response_Type | 1 | 603 | 0.02 | 36.06 | 0.40 | .530 | .00 |
| Modality | 1 | 603 | 0.06 | 5.40 | 6.73 | .010 | .01* |
| Gender x Response_Type | 1 | 603 | 0.36 | 36.06 | 6.09 | .014 | .01* |
| Gender x Modality | 1 | 603 | 0.04 | 5.40 | 4.06 | .044 | .01* |
| Response_Type x Modality | 1 | 603 | 0.00 | 5.40 | 0.23 | .631 | .00 |
| Gender x Response_Type x Modality | 1 | 603 | 0.00 | 5.40 | 0.12 | .734 | .00 |

*Note.* Asterisks indicate significant effects at p < .05; bold text indicate which higher-order effects had effect sizes that were medium-sized or larger. *df_Num_* indicates degrees of freedom numerator. *df_Den_* indicates degrees of freedom denominator. *SS_Num_* indicates sum of squares numerator. *SS_Den_* indicates sum of squares denominator. η^2^_p_ indicates partial eta-squared.
